# Differential Effects of Visual Feedback on Subjective Visual Vertical Accuracy and Precision

**DOI:** 10.1371/journal.pone.0049311

**Published:** 2012-11-12

**Authors:** Daniel Bjasch, Christopher J. Bockisch, Dominik Straumann, Alexander A. Tarnutzer

**Affiliations:** 1 Department of Neurology, Zurich University Hospital, Zurich, Switzerland; 2 Department of Otorhinolaryngology, Zurich University Hospital, Zurich, Switzerland; 3 Department of Ophthalmology, Zurich University Hospital, Zurich, Switzerland; University of British Columbia, Canada

## Abstract

The brain constructs an internal estimate of the gravitational vertical by integrating multiple sensory signals. In darkness, systematic head-roll dependent errors in verticality estimates, as measured by the subjective visual vertical (SVV), occur. We hypothesized that visual feedback after each trial results in increased accuracy, as physiological adjustment errors (A−/E-effect) are likely based on central computational mechanisms and investigated whether such improvements were related to adaptational shifts of perceived vertical or to a higher cognitive strategy. We asked 12 healthy human subjects to adjust a luminous arrow to vertical in various head-roll positions (0 to 120deg right-ear down, 15deg steps). After each adjustment visual feedback was provided (lights on, display of previous adjustment and of an earth-vertical cross). Control trials consisted of SVV adjustments without feedback. At head-roll angles with the largest A-effect (90, 105, and 120deg), errors were reduced significantly (p<0.001) by visual feedback, i.e. roll under-compensation decreased, while precision of SVV was not significantly (p>0.05) influenced. In seven subjects an additional session with two consecutive blocks (first with, then without visual feedback) was completed at 90, 105 and 120deg head-roll. In these positions the error-reduction by the previous visual feedback block remained significant over the consecutive 18–24 min (post-feedback block), i.e., was still significantly (p<0.002) different from the control trials. Eleven out of 12 subjects reported having consciously added a bias to their perceived vertical based on visual feedback in order to minimize errors. We conclude that improvements of SVV accuracy by visual feedback, which remained effective after removal of feedback for ≥18 min, rather resulted from a cognitive strategy than by adapting the internal estimate of the gravitational vertical. The mechanisms behind the SVV therefore, remained stable, which is also supported by the fact that SVV precision – depending mostly on otolith input - was not affected by visual feedback.

## Introduction

Internal estimates of the direction of gravity are essential for accurate and precise spatial orientation and navigation. Sensory input originating from both the otolith organs and the semi-circular canals and ascending along the graviceptive pathways [see [1] for review] is combined with input from skin and joint proprioceptors and vision at the level of the multisensory temporo-parietal cortex [2], [3]. The resulting signal provides a net estimate of earth-vertical. Among the various sensory systems involved in graviception, however, only the otolith organs directly sense the gravito-inertial force vector [4]. They provide the major input for perceiving static head-roll relative to gravity as measured, for example, by the subjective visual vertical (SVV) [see [5] for review]. Whereas healthy human subjects accurately indicate perceived vertical in upright position within ±2° [6], systematic errors are well known in roll-tilted positions. Whole-body roll-tilt requires central processing of body-roll angle β to rotate the visual line away from the body-longitudinal axis to earth-vertical for the SVV adjustment. At roll angles below 60°, variable over-compensation (E-effect) of angle β by a small and sometimes even non-significant amount has been reported [7], [8], [9], [10], [11]. With increasing head-roll angle roll over-compensation gradually decreases. At roll angles larger than 60° SVV follows a pattern of roll under-compensation (A-effect) [8], [9], [12], [13], first described by Aubert [14], peaking at 90 to 135° whole-body roll [13], [15].

In the presence of visual earth-vertical orientation cues, e.g. objects such as houses or trees, SVV adjustments are accurate and therefore no A- and E-effects are observed. Furthermore, tilted visual orientation cues induce deviations of perceived visual vertical in the direction of the tilted image [16], [17], [18]. However, when SVV alignments are performed in darkness (i.e. without visual orientation cues), A- and E-effects are present immediately [10], [19], [20]. These systematic, roll-angle dependent errors in estimated vertical are a typical feature of the luminous line paradigm. Using non-visual paradigms to indicate the perceived direction of gravity as by aligning a bar along vertical/horizontal [11], [21], [22], [23], by self-adjustments in the roll plane [12], [24] or by verbal reports of whole-body roll [9], [25], the A- and E-effects were greatly reduced or even eliminated.

Proposed mechanisms explaining these earth-vertical misestimations include central computational strategies based on otolith input and on optimization of the internal estimate of direction of gravity. Mathematical models linking otolith input to SVV vary widely, especially regarding assumptions made on the accuracy (i.e. the degree of veracity as reflected by the mean adjustment error) and the precision (i.e. the degree of reproducibility as reflected by the standard deviation or SD) of the otolith input. Based on anatomical observations by Rosenhall [26], Mittelstaedt [12] postulated an imbalance in the roll signal as a result of an unequal number of hair cells in the utriculus and sacculus, i.e. Mittelstaedt’s theory favors an otolithic origin of the perceptual roll misestimations. More recent SVV models were based on optimal observer theory [27], [28], [29], [30] and put the focus on the variability of the otolith input [31]. Bayesian models [8], [13] simulating SVV errors implemented an accurate, but noisy otolith signal. These models accurately reproduced observed A-effects (at angles >60°) and E-effects at large (>120–150° whole-body) roll angles by combining the otolith estimate of gravity with a bias vector, representing a prior expectation (“prior knowledge”) about the direction of gravity along the subject’s body-longitudinal axis. Furthermore, Bayesian models [8], [13] supported the hypothesis that SVV errors are a consequence of central computational mechanisms aiming to maximize the performance of verticality estimates near upright rather than an erroneous otolith source signal. Modulations of SVV precision as a function of head-roll were found to be related to the properties of the otolith sensors and to central computational mechanisms that are not optimally tuned for roll-angles distant from upright [13]. So far, experimental data as well as mathematical models have not been able to falsify that SVV accuracy and precision are independent variables. In fact, De Vrijer et al. [32] suggested a connection between SVV precision and accuracy by coining the term ‘accuracy- precision trade-off’, which implies that increased SVV precision at small tilts can only be obtained by decreased SVV accuracy at large tilts.

Previous attempts to modify A- and E-effects showed that both reducing proprioceptive input by water immersion [24], [33] and increasing the gravito-inertial force vector [34] had little effect, whereas rotating visual stimuli induced a significant shift in perceived visual vertical into the direction of the torsional optokinetic stimulus [35]. Bilateral vestibular deficits abolish the E-effect at small roll angles [36] and increase the A-effect at larger roll angles [37], [38], while impaired somatosensory function decreases the A-effect [39], [40], [41].

All afore mentioned paradigms aimed to better characterize the contribution of different sensory systems in generating an accurate and precise internal estimate of the direction of gravity. Most paradigms, however, have their limitations. E.g. studying SVV during water immersion to address the role of proprioception is technically demanding and limited in its use, and case studies with patients with bilateral vestibular deficits often show considerable heterogeneity with regards to the extent of the deficit, the underlying etiology and the disease duration. To bypass this and similar problems, modifying the reliability of specific sensory cues or adding additional cues may provide a means to study how the CNS integrates various sensory signals to obtain optimal estimates of the direction of gravity.

To better understand the mechanisms of roll over- and under-estimation we asked to which extent these adjustment errors can be modified behaviorally. Specifically, we hypothesized that providing visual feedback after each adjustment could be used to enhance the accuracy of the SVV as physiological adjustment errors (A−/E-effect) are likely based on central computational mechanisms. According to Krakauer and colleagues [42], behavioral performance can be improved through better state estimation (i.e., perceptual learning) and/or through better motor execution (i.e. motor learning), leading to “plasticity” [43]. Perceptual learning involves improving one’s ability, with practice, to discriminate differences in the attributes of simple stimuli [43]. Likewise, sensorimotor responses are re-calibrated by a continuous process of motor learning. With regards to these learning mechanisms, we hypothesized that the internal estimate of direction of gravity could be re-calibrated (or “shifted”) based on visual feedback indicating the participant’s adjustment error relative to gravitational vertical. During the adjustment trials, the motor system receives retinal input about the current line orientation in order to move it to the desired visual orientation. After turning the lights on, there will be a discrepancy between the desired line position and the actual (perceived) vertical. True adaptation is predicted to lead to an increase of SVV accuracy accompanied by a change of estimated direction of gravitational vertical, i.e. requires that the participants perceive their re-calibrated (and more accurate) adjustments as earth-vertical. Alternatively, the participants may - based on the visual feedback available - rather use a cognitive strategy [44] and consciously add a bias to the (unchanged) internal estimate of direction of gravity to better match true earth-vertical. While the first hypothesis implies that the subject perceives such optimized adjustments as earth-vertical, the second hypothesis predicts that the participant recognizes the optimized adjustment as roll-tilted as his/her internal estimate of gravitational vertical is unchanged.

Furthermore, we aimed to study whether changes in SVV accuracy have an impact on SVV precision also. We considered two alternative possibilities: 1) The mechanisms to optimize SVV accuracy might possibly hinder SVV precision, resulting in an increase of SVV trial-to-trial variability. This hypothesis takes into account the trade-off between SVV accuracy and precision described by De Vrijer and colleagues [32]. 2) Alternatively, the precision of SVV might remain unaffected as it mainly depends on the properties of the otolith afferent input and is modified by central computational mechanisms slightly only [13].

To test these hypotheses, we compared SVV adjustments in terms of errors (accuracy) and trial-to-trial variability (precision) in various whole-body roll-tilted positions with and without providing visual feedback after each adjustment. Visual feedback consisted of simultaneously displaying the direction of true earth-vertical and the orientation of the previous adjustment. Indeed roll under-estimations (A-effect) could be significantly reduced at roll angles ≥90° by providing visual feedback. This effect was found to outlast the removal of visual feedback and is rather related to a cognitive strategy than to a shift of the estimated direction of gravity as subjects perceived their modified SVV adjustments to be roll-tilted despite the fact that they were actually more accurate than their control adjustments. We therefore propose that the basics behind the SVV remained stable, which is also supported by the fact that SVV precision was not affected by visual feedback.

## Materials and Methods

Twelve healthy human subjects (3 females, 9 males; 24–53 yr old, mean age ±1 SD: 30±9) were studied. Two participants were familiar with the experimental setting; the other subjects were naïve.

### Ethics Statement

Written informed consent of all subjects was obtained after a full explanation of the experimental procedure. The protocol was approved by the local ethics committee (Ethics committee neurology, University Hospital Zurich) and was in accordance with the ethical standards laid down in the 1964 Declaration of Helsinki for research involving human subjects.

### Experimental Setting

All recordings were performed on a motor-driven turntable (Acutronic, Jona, Switzerland). Subjects were secured with a 4-point safety belt. The head was restrained in a natural straight-ahead position with a thermoplastic mask. Since the otolith organs are thought to have the greatest contribution on verticality estimation [13], [37], [45], are situated in the head, the subjects’ orientation in the roll plane will be referred as *head-roll orientation*, although roll movements on the turntable were whole-body, i.e., included both head and trunk. Turntable position, i.e. head roll position, was reached by turntable rotations about the naso-occipital axis with a triangular profile of 10°/s^2^ acceleration and deceleration. A remote control box allowed the subjects to rotate an arrow (covering the central 9.5° of the binocular visual field) projected on a sphere 1.5 m in front and to confirm adjustments. Myopic subjects wore their glasses or contact lenses.

### Experimental Paradigm

Nine head-roll orientations were studied in each subject, ranging from upright to 120° right-ear down (RED) in steps of 15°. We [13], as well as others [9], [15], [32], have not observed systematic differences in SVV responses for right-ear down roll orientations vs. left-ear down roll orientations previously, so we focused on RED in order to reduce the duration of data acquisition. All trials were collected in otherwise complete darkness. The arrow projection always started five seconds after the turntable came to a full stop and the arrow starting roll orientation was random within the entire 360° roll plane. In all sessions, subjects were instructed to adjust a luminous arrow within four seconds along the perceived gravitational vertical. The time limit of four seconds to complete the task ensured that subjects spent about equal time on the task in all conditions, which reduced potential time-dependent differences in arrow adjustment variability.

**Figure 1:**
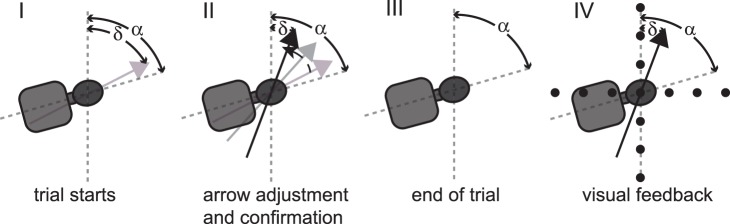
Illustration of a single SVV trial while the subject is tilted right-ear down (RED) by 75°, as indicated by angle α. At the beginning of each trial (A) the luminous arrow (in grey) is offset by angle δ. The subject then rotates the arrow towards perceived direction of vertical and confirms the adjustment when no further change is intended (illustrated by the arrow in black) (B). Then the arrow disappears (C) and either the next trial is started (control condition) or visual feedback of the adjustment is provided (D, test condition). For visual feedback, the room lights are turned on and both the arrow as adjusted by the subject and a grid oriented along earth-vertical and earth-horizontal become visible.

In each subject two sessions on either the same day (with a resting period of at least 4 hours between the two sessions) or on separate days were collected. Whereas the data collected in session 1 (standard SVV paradigm) served as a control, session 2 consisted of trials with the visual feedback. In seven of the 12 subjects an additional third session was recorded. The first half of this extra session contained trials with visual feedback and was immediately followed by the second half that consisted of trials with the standard SVV paradigm. Subjects who were invited to participate in the 3^rd^ session were selected based on the presence of a significant (p<0.05, ANOVA) A-effect in session 1. The goal of the third session was to study the time course of the adaptive effects on SVV accuracy and possibly SVV precision after removing the visual feedback again. In session 2 and the first half of session 3 visual feedback after each trial was provided. Therefore immediately after the trial a light illuminating the sphere was turned on and both the arrow position set by the subject and a cross consisting of two dotted lines along the earth-horizontal and along the earth-vertical axis at the level of the subject’s eyes were simultaneously visible for two seconds (see Fig. 1). In case of visual feedback, subjects were advised to take notice of the error made relative to the earth-vertical and earth-horizontal lines of the cross and to minimize this error in upcoming trials. For session 3 subjects were asked to retain the observed errors and compensate as much as possible in the following trials and also during the control trials that followed the trials with visual feedback. After sessions 2 and 3 subjects were asked whether they perceived their adjustments based on the visual feedback as earth-vertical (suggesting a shift of the internal estimate of direction of gravity) or not (implying a cognitive strategy while the internal estimate of gravitational vertical is unchanged).

In sessions 1 and 2, all nine head-roll orientations were studied. Data collection was split up into three blocks; each block consisted of 90 trials recorded in three different, adjacent head-roll orientations (e.g. upright, 15°RED and 30° RED), resulting in a total of 270 trials. We decided to group data recording in triplets of consecutive roll-tilt angles in order to facilitate learning in the visual feedback condition and to separate conditions with a tendency to E-effects (≤60°) from those with a tendency to A-effects (≥60°). Furthermore, pooling all nine roll angles studied would have required to run the entire session (lasting about 60 min) without breaks (as otherwise learning effects might have been lost again by turning on the light and allowing the subject to relax), increasing the risk of fatigue-related changes considerably.

As we were interested in whether increased accuracy of SVV adjustments outlasts the feedback period, we opted for roll angles where the A-effect is largest and most frequently found in session 3. Therefore, in session 3 data collection was restricted to the block with the three largest roll orientations (90, 105, and 120° RED). However, this block was run twice (once with and once without visual feedback). Before data collection subjects were given the opportunity to perform training trials. Both the order of blocks and the order of trials within each block were random. The single blocks lasted between 18 and 24 min (control condition) and between 21 and 27 min (test condition with feedback) in individual subjects.

Prolonged roll-tilts were shown to induce adaptation leading to drift of SVV errors [10], [19], [20] and ocular counterroll [46]. To minimize the effect of such temporal changes, whole-body roll position was changed after each trial. A short break with the lights turned on for ∼5 minutes was provided at the end of each block, terminating visual adaptation to the dark and allowing the subjects to relax and remove the mask. In session 3 there was no break between the two repetitions of the block (first run: with visual feedback, second run: identical roll angles but without visual feedback). During the post-adaptation period SVV adjustments were recorded over the duration of the second run of the block, i.e. during 18 to 24 minutes. A longer recording period after cessation of visual feedback was not feasible due to the discomfort for subjects in the roll-tilted positions.

Rotations with accelerations above the threshold of the SCC influence errors in dynamic SVV adjustments [47], [48]. For static SVV adjustments as used here we have previously checked for post-rotatory torsional ocular drift and nystagmus to quantify the contribution of SCC stimulation after the movement and demonstrated that average torsional eye velocity at the time when subjects confirmed arrow adjustments was small (0.10±0.06°/s) [49].

### Definition of Frequently Used Terms

According to the right-hand rule, clockwise (CW) shifts relative to the earth-vertical axis have positive signs and counter-clockwise (CCW) shifts yield negative signs. SVV *accuracy* denotes the degree of veracity, i.e. the difference between true earth-vertical and the actual SV setting (perceived earth-vertical) while SVV precision represents the degree of reproducibility, which is given by the inverse of the trial-to-trial variability.

### Data Analysis

Statistical analysis was performed using analysis of variance (ANOVA, Minitab, Minitab Inc., State College, USA). Tukey’s correction was implemented to compensate for multiple comparisons. Since trial-to-trial variability and adjustment errors in both paradigms (with and without visual feedback) depended on head roll, i.e. were dependent variables, Principal Component Analysis (PCA) was chosen to evaluate correlations. Standard correlations and regressions underestimate correlations and slopes when both components contain noise. PCA is equivalent to Orthogonal Linear Regression or Total Least Squares; it minimizes the perpendicular distances from the data points to the fitted model [50]. Multiple least square linear regression differs from PCA in that it implies that one variable, i.e. the independent variable, is known without error. Conversely, PCA adjusts for errors along all axes. As a measure of the goodness of fit we provide the R^2^-value. To estimate the sampling distribution of the slope of the fit obtained by PCA, we used bootstrapping. Data points were resampled 1000 times to compute the 95% confidence interval (CI). A correlation between the two dependent variables was considered significant whenever the 95% CI of the slope did not include zero.

In order to study the changes of adjustment errors - termed *SVV drifts* - over the course of a recording session (either with or without visual feedback), non-linear least square regression analysis (fit.m, Matlab, The MathWorks, Nantick, USA) with an exponential function was applied to individual roll-angles and subjects. Fitting provided the R^2^-value and the time constant (Tc) of the decay of the fitted exponential. To identify runs with exponential drift patterns, a goodness-of-fit (R^2^-value) of at least 0.2 was chosen as inclusion criterion. In these runs the impact of visual feedback on the drift was further analyzed by comparing median (±1 median absolute deviation or MAD) R^2^- and Tc-values in the two conditions. We hypothesized that by providing visual feedback, SVV drift is significantly reduced because of the visual reference, by which the subject notices an SVV drift.

## Results

**Figure 2:**
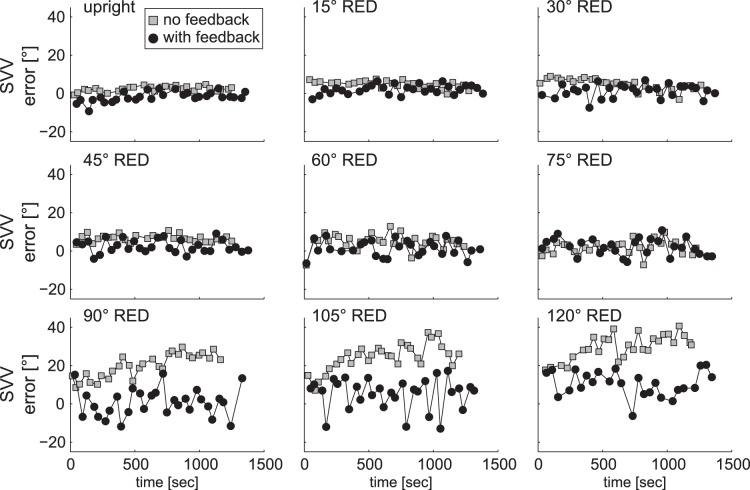
Single trial SVV adjustment errors are plotted against time for all head-roll orientations separately in a typical subject (DH) for both the control condition (no visual feedback, in grey) and the test condition (with visual feedback, in black). Compared to the control condition, adjustment errors relative to true earth-vertical were significantly reduced in the test condition at 90, 105 and 120° RED, while at the other roll angles no clear difference between the two conditions was noticeable.

SVV adjustments over time of a typical participant are depicted in Figure 2 both for control and test conditions. In the control condition (grey squares, Fig. 2) this subject showed a tendency to roll under-estimation (A-effect) at all roll-tilted orientations (being most prominent for 90°, 105°, and 120° RED). Adjustment errors were markedly decreased in case of visual feedback (black circles, Fig. 2). This decrease of errors appeared already over the course of very few trials.

### Adjustment Errors: with vs. without Feedback

**Figure 3:**
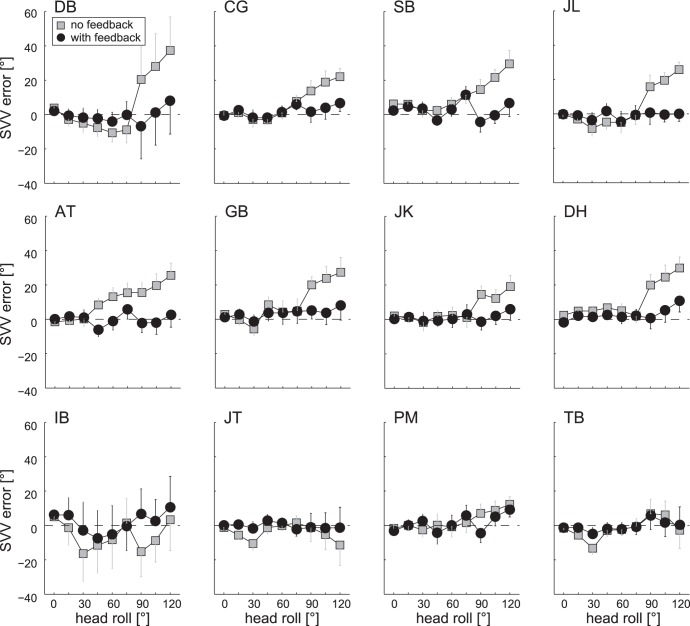
Individual average SVV adjustment errors for both the control condition (no visual feedback, in grey) and the test condition (with visual feedback, in black) are plotted against head-roll orientation in all subjects. The dashed horizontal lines refer to perfect SVV adjustments. While subjects in the first two rows all show a clear decrease in adjustment errors in the visual feedback condition compared to the control condition, subjects in the bottom row had either no A-effect in the control condition or showed no improvement by providing visual feedback.

Average individual adjustment errors (±1 SD) are depicted in Figure 3. Without visual feedback subjects aligned the luminous arrow accurately with earth-vertical in upright position (grand average ±1 SD: 1.3±2.8°), whereas in roll-tilted positions roll-angle dependent adjustment errors were observed. At head-roll angles below 60° RED small and variable roll over-compensation was found in six out of 12 subjects. These errors were maximal at 30° RED (grand average ±1 SD: 4.9±6.3°). For larger roll angles above 60° RED, however, increasing roll under-compensation (up to 18.2±14.6° at 120° RED on average) occurred in ten out of 12 subjects (see Fig. 3). By providing visual feedback after each trial in session 2, the pattern of adjustment errors considerably changed in individual subjects: in subjects that originally expressed roll over-compensation at small roll-tilts (30°, 45°) these errors decreased. For larger roll angles (>60–75°RED) – with ten out of 12 subjects showing various amounts of roll under-compensation – feedback resulted in a marked reduction or even elimination of these errors.

**Figure 4:**
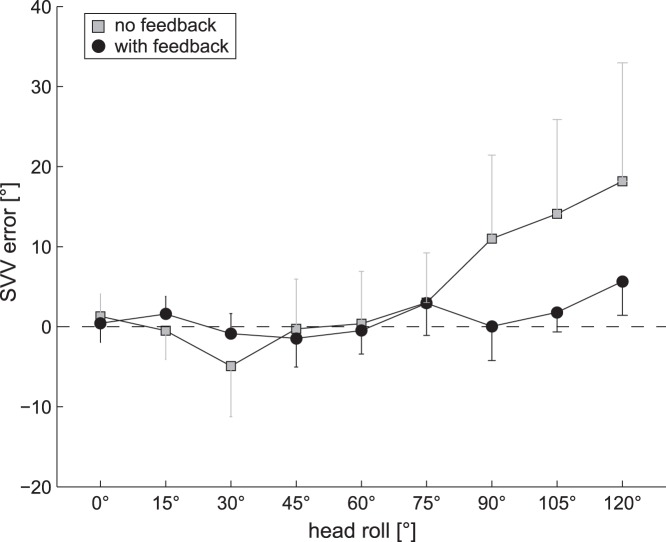
Grand average SVV adjustment errors (±1 SD) are plotted against head-roll for the control (in grey) and the test conditions (in black).

Grand averages of adjustment errors (±1 SD) are shown in Figure 4. In a 3-way ANOVA of individual average SVV accuracy (averaged unsigned error) there was a significant main effect for the condition (with vs. without visual feedback; F(1,22) = 190.7, p<0.001) and the roll orientation (F(1,22) = 40.5, p<0.001). In addition, there was a significant interaction between the conditions and the roll orientations (F(8,99) = 22.7, p<0.001). Pairwise comparisons of this interaction yielded significantly (p<0.001, Tukey-corrected) reduced unsigned errors due to visual feedback at roll angles of 90°, 105°, and 120° RED. At 30° RED, only a trend towards a reduction of the unsigned error (p = 0.098) was noted when providing visual feedback. At the other roll-angles tested, no differences between the adjustment errors in the two conditions were noted. No main effect for the direction of luminous-arrow rotation on adjustment errors (F(1,22) = 0.06, p = 0.810) was apparent. Therefore for further analysis of SVV accuracy, trials with clockwise and counter-clockwise visual arrow rotations were pooled.

### Drift of SVV Over Time

When fitting an exponential function to the individual runs in the control condition, an R^2^-value of 0.2 or larger (see methods section) was found in 45% of runs (49/108), with a median R^2^-value of 0.38 (±0.14; 1 median absolute deviation or MAD) and a median time constant of 17.6 (±4.9 min). Such exponential SVV drift was noted in at least half of the subjects at 15°RED (6/12, 50%), 30°RED (10/12; 83%), 90°RED (6/12, 50%) and 105°RED (8/12; 67%) while in all remaining roll orientations 3 to 5 subjects fulfilled the criteria. Median absolute drift amplitudes for those subjects with significant drift over the recording period (18 to 24 min in individual subjects) ranged between 9 and 11° for all head-roll angles larger than 15°RED studied with the exception of 75°RED (drift amplitude = 18°, however, based on a sample size of n = 3 only). The exponential SVV drifts were significantly (p<0.001, Fisher’s exact test) more likely to be increasing (78%, 38/49 runs) than decreasing (22%, 11/49 runs). For specific head-roll orientations, the median amplitudes of the individual exponential drifts were significantly larger than zero at 90°RED (p = 0.03, signrank.m) and at 105°RED (p = 0.02), which indicates a preference of A-effects to increase. At 30°RED, however, only a trend (p = 0.06) towards an increased E-effect was found. For all other head-roll angles no such preference was noted at the group level.

Providing visual feedback resulted in a significant (p<0.001, signrank.m) decrease of the goodness-of-fit (R^2^ = 0.09±0.07; median ±1. MAD) in those runs that presented with an R^2^-value ≥0.2 in the control condition. Likewise the median Tc (±1 MAD) of decay significantly (p<0.001) increased with visual feedback (31.8±21.4 min), exceeding the recording time per run (21 to 27 min). Furthermore, visual feedback reduced amplitudes of SVV drift in all head-roll orientations. In 15 cases, however, significant exponential drift was noted in the test condition only, while in the corresponding control condition this was not the case.

### SVV Precision

Three-way ANOVA yielded no main effect of the direction of arrow rotation for the precision (i.e. the inverse of the trial-to-trial variability) of SVV adjustments, therefore trials with CW and CCW arrow rotations were pooled for further analysis. Unlike the adjustment errors, which were found to be significantly reduced at large head-roll angles when providing visual feedback, SVV precision did not show a main effect for the trial condition (without visual feedback vs. with visual feedback) (F(1,22) = 2.46, p = 0.118). Furthermore, no significant interactions between the different factors (trial condition, direction of arrow roll rotation, whole-body roll orientation) were observed.

**Figure 5:**
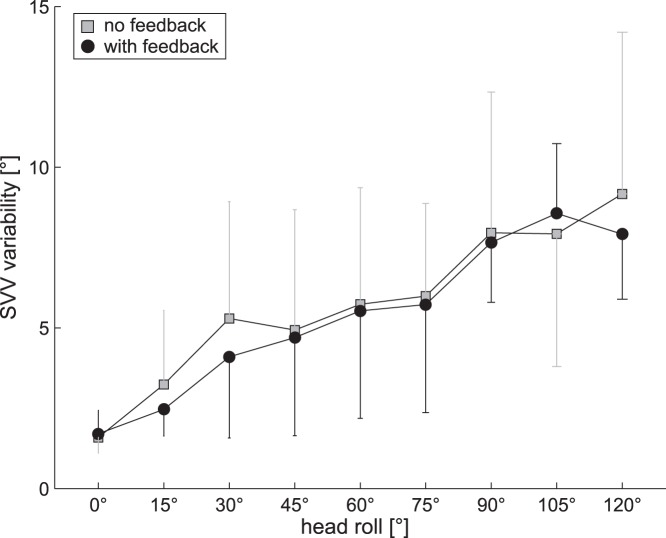
Grand average (±1 SD) trial-to-trial variability (pooled from all 12 subjects) is plotted against head-roll orientation both for the control (grey) and the test (black) condition.

In both trials with and without visual feedback SVV precision significantly depended on the head-roll orientation (F(8,99) = 55.73, p<0.001), with larger variability values at larger head-roll orientations, as indicated in Figure 5, illustrating the grand average SVV trial-to-trial variability within subjects. This pattern is in agreement with previous SVV studies [8], [13], [15], [45] and could be explained by a decreasing efficiency of the otolith afferents and by central computational mechanisms providing optimal tuning of the otolith signal near upright position only [13].

### Temporal Evolvement of SVV Accuracy After Removal of Visual Feedback

**Figure 6:**
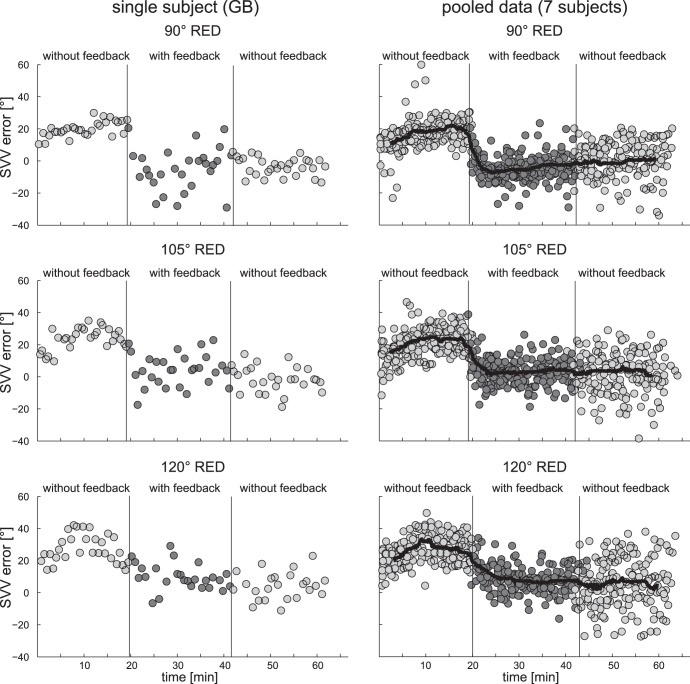
Comparison of adjustment errors obtained with distinct feedback conditions, split up in three different blocks (first without visual feedback, second with visual feedback, and third, immediately after the previous block without pause, again without visual feedback) are plotted against time for head-roll orientations of 90°RED, 105°RED and 120°RED. While the left column shows single subject data (subject GB), the right column illustrates the pooled individual trial data from all subjects (n = 7). Trials without visual feedback are in light grey, trials with visual feedback in dark grey. A running median (solid black line, window size: 50 samples) is also depicted. Note that the first block (without visual feedback) originates from the control session (session 1), while the second and third blocks were obtained in session 3.

In seven out of 12 subjects a third session consisting of a first block with visual feedback immediately followed by a second block without visual feedback was obtained at roll angles of 90, 105 and 120° RED. All subjects selected for session 3 previously had shown substantial A-effects in the control session without visual feedback. After a full block of trials with visual feedback a sustained reduction of adjustment errors was noted in the consecutive block without visual feedback as shown in Figure 6. Statistical analysis (ANOVA) of individual average SVV adjustments over the three sequences yielded a significant main effect of the condition (no visual feedback vs. visual with feedback vs. immediately after visual feedback) in all three head-roll orientations studied. As the different head-roll positions were studied separately, Bonferroni correction was applied to correct for the number of tests (n = 3). Multiple comparisons showed a significant decrease (p≤0.001) in absolute adjustment errors both with visual feedback and when repeated immediately after visual feedback in all three head-roll orientations. This suggests that the feedback-driven improvement of adjustment accuracy leads to a prolonged change in how subjects perform the task also after removal of visual feedback for a time period of at least 18 to 24 minutes without obvious decreases during this period.

## Discussion

Perception of gravity as measured by the subjective visual vertical (SVV) results in a well-known pattern of misestimations of the SVV in head roll-tilted positions. It was suggested that these errors reflect a strategy of the brain to optimize the precision of adjustments near upright [8], [13]. Here we studied how changing the SVV task from an open-loop condition (without visual feedback) to a closed-loop condition (with visual feedback) affects performance. Our underlying hypothesis was that trial-by-trial visual feedback leads to increased accuracy of SVV adjustments, either by adaptation of by a cognitive strategy. The findings reported here confirm that the visual feedback paradigm applied results in a significant (p<0.001) reduction of roll under-compensation at head-roll angles of 90° and larger, thus almost eliminating the A-effect seen in the control condition (i.e. without visual feedback). For head-roll angles up to 60°, where slight roll over-compensation in SVV paradigms without feedback is known [9], [11], providing visual feedback resulted in subtle and non-significant reductions in the E-effect, being most effective at a head-roll angle of 30°.

The inter-individual variability – representing a measurement of the homogeneity of adjustments within a population – decreased considerably when providing visual feedback, which further underlines the modulatory effect of visual feedback on the adjustment performance. Unlike the significant reduction in absolute adjustment errors observed when providing visual feedback, SVV precision remained unchanged. In the control condition we observed slow, but significant exponential drift (median Tc = 17.6 min) of SVV adjustments over time in 45% of the runs. This drift resulted significantly (p<0.001) more likely in a decrease of SVV accuracy (78% vs. 22% of cases, decrease vs. increase of SVV accuracy) and increased E-effects (at small roll angles) and A-effects (at large roll angles). In the visual feedback condition a reduction of exponential drift, as reflected in a significantly longer Tc and reduced drift amplitudes, was noted.

### Accuracy of SVV Improved Significantly with Visual Feedback

Our findings confirm the hypothesis that visual information about task performance can be implemented by the subjects to improve the accuracy of SVV adjustments in future trials – even if they are performed in alternating order in varying head-roll orientations relative to gravity. Similar observations have been made for the subjective postural vertical by Clark and Graybiel [51]. Providing the subject with true earth-vertical after each adjustment of perceived postural vertical they found decreasing adjustment errors as well.

The elimination of the A-effect by our closed loop SVV paradigm further supports the hypothesis that the A-effect is of central origin and not due to an erroneous otolith source signal. Such a central mechanism can explain why the A-effect can be modulated by higher cognitive strategies as shown here, while the precision of SVV adjustments – presumably depending mainly on the properties of the otolith afferents - remains unchanged when providing feedback. It has been hypothesized, that the A- and E-effect are side effects of the brain’s strategy to optimize the precision of internal estimates of the direction of gravity in whole-body roll positions near upright [8], [13]. As a result of this strategy, central computational mechanisms are not optimally tuned for roll-angles distant from upright [13]. Furthermore, the presence of the A- and E-effect seems to be bound to visual input. By use of non-visual paradigms to indicate the perceived direction of gravity as the subjective haptic vertical or horizontal [11], [21], [22], [23], the subjective postural vertical [12], [24] and verbal reports of whole-body roll [9], [25], the A- and E-effects could be significantly reduced or even disappeared completely.

The lack of significant changes at smaller head-roll angles is likely related to the more subtle and variable presentation of the E-effect in our study. Similar observations were reported in previous studies, showing that the E-effect varies considerably between subjects, ranging from clear roll over-compensation (E-effect) of up to 6° (peaking around 30–45° roll orientation) to accurate estimates of vertical/horizontal [9], [10], [11], [13], [15], [32].

We found the reduction of adjustment errors to remain significant after removal of visual feedback, suggesting a prolonged effect of the visual feedback paradigm. This finding is novel and underlines learning induced by the closed loop paradigm. We will consider different kinds of learning, including perceptual learning (optimizing the use of sensory input to improve future adjustments) and motor learning (improving motor execution) and higher cognitive strategies. The task imposed here does not require skillful hand/arm movements; the motor system is rather guided by visual feedback to move the line to the desired visual orientation. Motor learning therefore is unlikely to lead to improved task performance here. It is rather a visually perceived discrepancy between the desired (earth-vertical) line roll orientation and the actual adjustment position that facilitates learning, which is in accordance with the concept of perceptual learning. Perceptual learning has been proposed to reflect implicit memory [43] and involving subconscious [52] skill improvements. Conscious awareness of the adjustment error, as it is the case in our paradigm, however, does not preclude it from leading to perceptual learning. But it does make the distinction between adaptation and higher cognitive strategies more difficult. We therefore asked all participants how they had completed the task when visual feedback was available. Subjects confirmed being aware of their adjustment errors and all but one subject reported a strategy consistent with adding an offset to the percept of earth-vertical to generate more accurate SVV adjustments, which, however, they did not perceive as earth-vertical. This observation favors a cognitive strategy over perceptual learning and suggests that the internal estimate of direction of gravity was not modified by the visual feedback paradigm used here.

In light of adaptational changes of sensorimotor responses in order to maintain optimal performance found in many systems [43], [53], [54], [55], [56], [57], [58], lack of perceptual learning in our study was unexpected and deserves further attention. Possibly, the feedback stimulus provided was not sufficient to induce adaptation or the number of repetitions with feedback was too small. Other paradigms used to successfully induce perceptual learning in vision research provided repetitive sessions over several days [54], while only one feedback session lasting less than 30 minutes was run here. However, depending on the experimental conditions, brief (<10 min) periods of training are sufficient to induce perceptual learning [44]. Therefore, the relatively short feedback period does not necessarily exclude the possibility of sufficient training. The visual feedback about the size of the adjustment error was perceived by all subjects well and was straight-forward. However, perceived direction of gravity is a highly integrated estimate based on input from various peripheral sensors and prior knowledge [13]. Providing additional input through the visual system only while keeping the other sensory (e.g. proprioceptive and otolithic) input unchanged might not have sufficient weight to bias the perceived direction of gravity in future trials.

Due to the strain of lying in a side position for a prolonged period, the post-feedback part of session 3 was limited to 18 to 24 minutes. As the decrease of adjustment errors achieved during the visual feedback period remained stable over this post-feedback period, we cannot make any predictions about the further temporal evolvement of this effect and the associated time constant. While increases of the recording time may help determine the time constant of decay, fatigue will also play an increasing role, potentially confounding a fading learning effect.

Using an exponential function to fit the drift pattern, the internal estimate of perceived vertical was not stable over time in almost half of all runs. Others previously reported drifts for repetitive adjustments in upright position and during prolonged roll-tilts in the absence of any visual feedback [10], [19], [59], [60]. In earlier work we proposed that changing the subject’s head-roll orientation after each trial may prevent adaptation to a given head-roll angle and therefore may reduce drift [13]. The data presented in this study suggests that changing the subject’s roll position after each trial by 30° or less may not be sufficient to remove drift of perceived vertical over time. Therefore larger shifts in head-roll orientation between individual trials might be required to minimize adaptation to a given head-roll orientation over time. Proposals for the origin of these drifts include adaptation in the involved sensory systems (i.e. proprioception and the otolith organs) [10], long-range serial dependence (termed 1/f beta noise [61]) [60], and central compensational mechanisms [60]. Compared to the control conditions, exponential drift occurred in a smaller fraction of subjects and was of smaller amplitude in the visual feedback conditions in the study presented here. By providing feedback, errors emerging in an open loop paradigm can be counteracted, as shown here for visual feedback.

### The Precision of Adjustments Remains Unaffected by Visual Feedback

We discussed two distinct hypothetical mechanisms how accuracy and precision of SVV adjustments could be linked in the introduction. While the first hypothesis predicted a decrease in SVV precision as SVV accuracy increases (accuracy-precision trade-off [32]), the second hypothesis considered accuracy and precision of SVV adjustments as mainly independent, i.e. an increase in SVV accuracy does not necessarily affect SVV precision. Based on our experimental data, showing that SVV precision is not significantly changed by visual feedback while SVV accuracy is significantly increased at angles ≥90° RED, we suggest that the precision of SVV adjustments does not relate to the size of the adjustment error. For the changes in SVV accuracy achieved by the visual feedback paradigm used here, therefore a trade-off between accuracy and precision seems unlikely, as this hypothesis would predict a decrease in precision when increasing the accuracy. Lack of changes in SVV precision in the visual feedback conditions is likely related to the cognitive strategy used by the subjects. If indeed an offset (derived from visual error feedback from previous trials) is added to the perceived direction of gravity to improve SVV accuracy, trial-to-trial variability will only be affected if the size of this offset varies significantly from trial to trial. The change in SVV accuracy achieved by visual feedback, however, reached a plateau within few trials and therefore rather supports a fairly constant offset (assuming that the internal estimate of direction of gravity remains stable).

### Conclusions

Visual feedback indicating the error between perceived and true direction of gravity resulted in significantly improved SVV accuracy at roll angles ≥90° whereas SVV precision remained unchanged at all roll angles studied. This effect was found to persist for at least 18 to 24 minutes after removal of visual feedback and is most likely related to a cognitive strategy rather than to an adaptational shift (i.e. motor or perceptual learning) of the estimated direction of gravity. We conclude that roll under-estimation (A-effect) can be modulated cognitively both during and immediately after providing visual feedback. The dissociation between the reduced mismatch (as reflected by the decrease in adjustment errors relative to true earth-vertical) and the unchanged percept of direction of gravity, however, speaks against the presence of adaptation induced by the visual feedback paradigm used in this study. It rather suggests that the central computational mechanisms (based on sensory input and prior knowledge) providing the internal estimate of direction of gravity remained stable, which is also supported by the fact that SVV precision – depending mostly on otolith input - was not affected by visual feedback. Furthermore, our data suggests that shifts in head-roll orientation by 30° or less after each trial may not be sufficient to avoid adaptation to prolonged static roll-tilted positions and consecutive drift in perceived vertical. Larger shifts or even return to upright position and a natural visual surrounding may be needed to eliminate such drifts.
